# Belt restraint reduction in nursing homes: design of a quasi-experimental study

**DOI:** 10.1186/1471-2318-10-11

**Published:** 2010-02-25

**Authors:** Math JM Gulpers, Michel HC Bleijlevens, Erik van Rossum, Elizabeth Capezuti, Jan PH Hamers

**Affiliations:** 1 School for Public Health and Primary Care (Caphri), Department of Health Care and Nursing Science, Maastricht University, Maastricht, the Netherlands; 2 Zuyd University of Applied Sciences, Research Centre on Autonomy and Participation of chronically ill patients, Heerlen, the Netherlands; 3 New York University, Hartford Institute for Geriatric Nursing, New York, USA

## Abstract

**Background:**

The use of physical restraints still is common practice in the nursing home care. Since physical restraints have been shown to be an ineffective and sometimes even hazardous measure, interventions are needed to reduce their usage. Several attempts have been made to reduce the use of physical restraints. Most studies used educational approaches and introduced a nurse specialist as a consultant. However, the success rate of these interventions has been inconsistent. We developed a new multi-component intervention (EXBELT) comprising an educational intervention for nursing home staff in combination with a policy change (belt use is prohibited by the nursing home management), availability of a nurse specialist and nursing home manager as consultants, and availability of alternative interventions. The first aim of this study is to further develop and test the effectiveness of EXBELT on belt restraint reduction in Dutch psychogeriatric nursing homes. However, the reduction of belts should not result in an increase of other restrictive restraints (such as a chair with locked tray table) or psychoactive drug use. The overall aim is an effective and feasible intervention that can be employed on a large scale in Dutch nursing homes.

**Methods and design:**

Effects of EXBELT will be studied in a quasi-experimental longitudinal study design. Alongside the effect evaluation, a process evaluation will be carried out in order to further develop EXBELT. Data regarding age, gender, use of physical restraints, the number of falls and fall related injuries, psychoactive drug use, and the use of alternative interventions will be collected at baseline and after four and eight months of follow-up. Data regarding the process evaluation will be gathered in a period of eight months between baseline and the last measurement. Furthermore, changing attitudes will become an important addition to the educational part of EXBELT.

**Discussion:**

A quasi-experimental study is presented to investigate the effects of EXBELT on the use of belts on wards in psychogeriatric nursing homes. The study will be conducted in 26 wards in 13 psychogeriatric nursing homes. We selected the wards in a manner that contamination between control- and intervention group is prevented.

**Trial registration:**

(NTR2140)

## Background

The use of physical restraints still is common practice in the nursing home care of older people with dementia. Physical restraints are defined as any limitation in an individual's freedom of movement [1] and includes those worn by the person (belt, chest, and arm/leg), those attached to beds (full-enclosure bedrails) or chairs (locked table). Although reports of restraint prevalence internationally varies from 15 to 66% [2,3], ranges of restraint prevalence in Dutch nursing homes is between 41 to 64% [2]. Recent prevalence measures in the Netherlands have shown that 10% to 14% of nursing home residents are restrained with belts [4,5].

Staff report that physical restraints are used to prevent falls [6-8]. The accumulating evidence that restraint reduction does not lead to an increased number of falls or fall-related injuries [9-11] and that restraint use can result in falls and problems with balance and coordination [6,12,13], call into question the continued use of these devices as "safety measures". Restraints have other known negative physical, psychological and social consequences for older persons. Both prolonged and short periods of physical restraint use are associated with pressure sores, loss of muscle strength and endurance, joint contractures, incontinence, demoralization, humiliation feelings of low self worth, depression, aggression and impaired social functioning [6,14,15]. Evans and colleagues [12] conducted a systematic review of physical restraint use in acute and residential health care facilities, and documented that the use of physical restraints (including belts), increases the risk of death, serious injury, as well as the length of hospital stay. Older adults report negative feelings about the restraint experience such as discomfort and indifference [16]. The use of restraints may also indicate a failure to address the real needs of the residents. Since physical restraints have been shown to be an ineffective and sometimes even hazardous measure, interventions are needed to reduce their usage.

Several attempts have been made to reduce restraint use in clinical practice [17-22]. Most interventions used educational approaches, aiming to improve nursing staff knowledge and confidence to avoid physical restraints and to use alternative measures that target the resident's underlying problems [18,19,21,22]. These intensive training sessions were delivered to staff by a nurse specialist provided to the nursing home as a consultant from the study team. The success rate of these interventions differs between countries; a successful educational intervention in the USA [18] proved to be ineffective in the Netherlands [19,23,24]. It is unclear whether these contradictory results can be explained by cultural differences, differences in health care systems, or difference in educational level of nursing staff in nursing homes between the USA and the Netherlands. The results of a recent study among Dutch, German and Swiss nursing staff indicate that opinions and attitudes towards physical restraints hinder attempts to reduce restraint use [25]. In this study, almost all nursing staff assessed the use of physical restraints in their clinical practice as appropriate. Moreover, Dutch nursing staff consistently assessed restraint measures as less restrictive than German and Swiss nursing staff and reported less discomfort in using restraints [25]. Furthermore, this and other studies indicate that the availability of alternative interventions is essential for effective restraint reduction [19,23-25]. For instance, in the study by Huizing and colleagues it was reported that the availability of some new and rather expensive alternative measures was limited [24]. Finally, there are indications that legislation influences the use of restraints; the success of the educational intervention in the USA has also been supported by a nursing home law that led to national nursing home regulations (OBRA '87) that discourage restraint use [26,27]. In the Netherlands, the secretary of state of the Ministry of Health, Welfare and Sports in 2009 has introduced a bill in Parliament ("Wet Zorg en Dwang") that regulates the use of physical restraints in people with dementia in general and belts in particular. According to this bill, the usage of belts to prevent falls will no longer be allowed [28]. The proposed changes in legislation provide an opportunity to develop a multi-component intervention tailored to the Dutch nursing home environment that will assist facilities in meeting this new requirement.

Among restraints used in Dutch nursing homes, belts are the most restrictive measure [4,19] therefore our intervention program, named EXBELT, primarily focuses on belt use reduction [25]. The EXBELT intervention includes four components: (1) promotion of institutional policy change that discourages belt restraint use, (2) education, (3) consultation by a nurse specialist, and (4) development and availability of alternative interventions. Cultural differences in staff opinions is an important consideration for the development of effective interventions. The educational component (including strategies for changing attitudes) for nursing home staff (physicians, nurses, paramedical staff and psychologist) is based on the intervention developed by Evans and colleagues [18] and Huizing and colleagues [19,23-25] that is customized for Dutch nursing home staff.

In 2007, EXBELT was developed and piloted in one nursing home ward [29]. The results of this pilot study were very promising. At baseline, 12 belts in 30 residents were used. After one month follow up, no belt was used, but after three and nine months follow up 1 belt was used. This reduction in belt use did not result in either an increase in the number of falls and related injuries or the use of other restrictive measures such as chairs with a locked tray table or psychoactive medication. Belts were replaced with, resident-centered interventions, such as movement and balance training, lower beds, hip protectors, extra supervision and monitoring devices (video camera, sensor mat, and infrared alarm systems). The recent expansion of the pilot (in 2008) to other wards in the same nursing home has shown similar results. However, this home does not represent a typical Dutch facility since it is considered as 'best practice' regarding restraint reduction initiatives and research. Thus, further testing of the EXBELT intervention is needed on wards in other nursing homes prior to widespread dissemination.

## Study aim and research questions

The first aim of this study is to further develop and test the effectiveness of a tailored multi-component intervention program (EXBELT) on belt restraint reduction in Dutch psychogeriatric nursing homes. However, the reduction of belts should not result in an increase of other restrictive restraints (such as a chair with locked tray table) or psychoactive drug use. The overall aim is an effective and feasible intervention that can be employed on a large scale in Dutch nursing homes. We translated the aims into the following eight specific research questions:

1. Does a tailored multi-component intervention (EXBELT) result in the reduction of belts in nursing homes?

2. Does EXBELT prevent the use of belts in newly admitted residents?

3. Does EXBELT reduce the use of other types of physical restraints?

4. Does belt elimination result in an increase of falls and fall related injuries?

5. What resident centered alternative interventions are used in EXBELT?

6. What is the opinion of nursing home staff, management and residents' relatives about EXBELT and the effectiveness of alternative interventions?

7. What are indicators for successful or unsuccessful implementation of EXBELT?

8. What improvements (related to content, organization and monitoring) are necessary to optimize the effect of EXBELT?

## Methods/Design

### Design and sample

**Figure 1:**
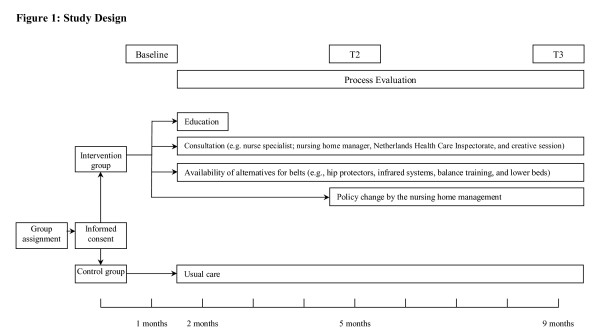
**Study Design**.

Effects of EXBELT will be studied in a quasi-experimental longitudinal study design. Alongside the effect evaluation, a process evaluation will be carried out in order to further develop EXBELT. Figure 1 shows the design of the study presented. After contacting seven Dutch nursing home associations (networks of nursing homes) in order to assess whether they would be interested to participate in our study, four nursing home associations, located in three regions in the Netherlands (Zuid-Limburg, Midden Limburg/Zuid-Oost Brabant en Zuid-Holland) contacted the EXBELT research group to participate in the current study. To participate, the prevalence of belt use on psychogeriatric nursing home wards had to be at least 10%. Wards are excluded if the unit is dedicated to residents with Korsakoff's, if far-reaching reorganizations and/or constructional renovations will be implemented, and if participating in other studies and/or projects aimed at the reduction of restrain use. The total study sample comprises four nursing home associations, 13 nursing homes with a total of 26 psychogeriatric wards. The 26 wards were assigned to either the intervention or control group. Assignment to either to intervention or control groups was carried out by the research team. Since no randomization took place, allocation was based on avoidance of contamination bias. Overlap of nursing home staff between the intervention and control wards was averted. In addition, based on the geographical location of the participating wards, wards from each of the four nursing associations that were situated closely together were allocated to the same group. The wards allocated to the control group will receive care as usual, while the wards allocated to the intervention group will receive the EXBELT program.

The management boards of the four participating nursing home associations agreed with the implementation of the EXBELT program. After allocation of the wards to intervention and control groups, written information about the study will be given to the residents' legal representatives, and written informed consent will be obtained from the legal representatives of the nursing home residents in order to include those residents in the study. Data will be collected at baseline (T1), and four (T2) and eight (T3) months after baseline.

The study design and protocol were approved by the Medical Ethics Committee of the University Hospital Maastricht and Maastricht University. In addition, local Ethical Committees of participating institutions have given their consent to the protocol and procedures.

### Sample size considerations

Sample size calculations are based on the primary outcome measure for residents: proportion of residents using a belt. We expect a reduction of 50% in belt use in the intervention group and no changes in use in the control group. Based on a significance level α of 0.05 (two sided) and a power of 60%, 216 residents are needed in each group in the analyses. Taken into account an informed consent rate of 80% and a drop-out rate of 25%, 720 psychogeriatric nursing home residents have to be selected at the start of the study.

### Intervention program

EXBELT is a multi-component intervention that comprises a policy change, in combination with an educational intervention for nursing home staff and consultation by a nurse specialist, and the use and availability of alternative interventions. The four key components of EXBELT are:

1. Implementation of institutional policy change that prohibits belt restraint use including communication of the policy change to:

a. nursing home staff;

b. residents' relatives.

2. Intensive educational intervention program for nursing home staff (nursing home physicians, nurses, paramedical staff, psychologists and ward managers) that address changing staff attitudes. Key parts are:

a. focus on safely reducing belts with the understanding that nursing homes never can guarantee no falls or related injuries;

b. taught by a nurse specialist during three small-scale meetings, each lasting three hours, over a three week period;

c. small-scale meetings attended by nursing home staff from different wards;

d. a 90-minute educational session directed toward all nursing home staff of each intervention ward after the three small-scale meetings were delivered;

e. one specific module focused on strategies for changing attitudes such as shifting perspectives [30,31].

3. Consultation:

a. the nurse specialist who delivered the educational program will provide on-site consultation to individual nurses on the intervention wards regarding challenges in reducing restraints for specific resident cases [18];

b. a nursing home manager and the Netherlands Health Care Inspectorate (IGZ) will be available as a consultant to nursing home managers and clinical staff for individual cases, as needed;

c. representatives of the nursing staffs, product developers, and the research team will discuss problem cases in a creative session.

4. Development and availability of alternative interventions:

a. directors of the involved nursing homes will provide resident centered alternative interventions available including hip protectors, infrared systems, balance training, exercise, special pillows and lower beds;

b. the nurse specialist stimulates en facilitates decision-making regarding alternative interventions by multidisciplinary team;

c. the nurse specialist encourages the use of alternative interventions.

## Measures

### Effect evaluation

The primary outcome measure of the effect evaluation is the use of belts. Belt use will be measured at baseline, T2 and T3 using the observation tool developed by Huizing and colleagues [19,23,24]. Belts per resident will be recorded as present or absent. The use of belts will be measured by a single trained observer, who is blinded to the group assignment, on four times during a 24-hour period (morning, afternoon, evening and night). The day each of the participating wards is visited will be unannounced in order to prevent any artificial removal of belts by nursing home staff.

Secondary outcome measures include other types of physical restraints (e.g. bilateral full-enclosure bedrails, deep or overturned chairs, chairs with a locked tray table, chairs on a board), psychoactive drug use, number of falls and fall-related injuries, the use of alternative interventions, cognitive level, activities of daily living (ADL)-status, ADL-dependency, and mobility. Physical restraint use will be measured at baseline, T2 and T3 using the same observation tool to measure the usage of belts developed by Huizing and colleagues [19,23,24]. Types of physical restraints per resident will be recorded as present or absent. The day each of the participating wards is visited will be unannounced in order to prevent any artificial removal of physical restraints by nursing home staff. Data on psychoactive drug use will be collected at baseline, T2 and T3 using the residents' medical records. Falls and fall-related injuries will be recorded retrospectively (three months preceding each of the three measurements, using the register of falls that Dutch nursing homes are required to maintain [32]. The use of alternative interventions used will be monitored continuously, using a report chart (addressing type of intervention) that will be filled out by the nursing staff. Data concerning cognitive level, activities of daily living (ADL)-status, ADL-dependency, and mobility will be collected only for those residents who are restrained by a belt. Cognitive status will be measured using the Cognitive Performance Scale [33]; ADL-status will be measured using the ADL Self-performance Hierarchy[34]; ADL dependency will be measured using the Barthel index [35]; and mobility will be measured using a mobility scale developed from MDS items [23]. Agitation will be measured using the Cohen-Mansfield Agitation Index-short form [36,37]. In addition, socio-demographic characteristics will be assessed at baseline, T2 and T3 for all residents participating in the study: age and gender.

### Process evaluation

For conducting the process evaluation, other samples will be recruited in comparison with the effect evaluation. Depending on the study question, residents' relatives, nursing home staff, educators (nurse specialists), nursing home management, and the Netherlands Health Care Inspectorate (IGZ) will be invited to participate in different parts of the process evaluation.

The process evaluation will monitor the content and feasibility of the intervention program. Data regarding the process evaluation will be gathered regarding the eight month period between baseline and T3. First, to investigate the opinion of nursing home staff, the nursing home management and the residents' relatives about EXBELT and the effectiveness of alternative interventions, structured interviews will be used. Second, intervention fidelity, including the dose delivered, and dose received [38], will be obtained by conducting interviews with nursing home staff, nursing home management, educators and consultants. In addition, checklists and observation forms will be used to document fidelity of the intervention across nursing home units assigned to the intervention group. Finally, to examine the influence of the EXBELT on attitudes and opinions regarding restraint use, we will measure attitudes of nursing home staff using the MAQ [25] at baseline, after the education program and at the end of the project.

### Data analysis

Comparability between the intervention and control groups will be assessed at baseline to check for differences between the two groups on socio-demographic characteristics (age and gender). Outcomes at T2 and T3 will be compared between the intervention and control groups by both univariate and multivariate techniques. Data resulting from the effect evaluation will be primarily analyzed according to the intention-to-treat principle, i.e., including all participants with valid data, regardless of whether they remained in the setting which they were measured at baseline. Subsequently, the results of the intention-to-treat analysis will be compared with the results of a per-protocol analysis, to assess whether protocol deviations have caused bias. In all analyses effect estimates will be adjusted for baseline differences. Dropouts and losses-to-follow up will be described. Data resulting from the process evaluation will mainly be analyzed by means of descriptive techniques.

### Study progress

In November 2008 the Medical Ethics Committee of the University Hospital Maastricht and Maastricht University has approved the study design and protocols. In December 2008 we started the selection of the nursing homes. The informed consent procedure began in February 2009. Representatives of the residents received written information and were asked to give written informed consent for the use of personal data on the residents in the study.

Baseline measurements followed in February and March and the implementation of EXBELT was started in March and April. The last follow up measurements are planned for the end of 2009 (effect evaluation) and early 2010 (process evaluation). Analyses of the data and dissemination of results are planned for 2010.

## Discussion

This paper presents the design of a quasi-experimental study, which aims to explore the effect and feasibility of an intervention program (EXBELT) that aims to reduce the use of belts in Dutch psychogeriatric nursing homes. Some methodological and practical drawbacks, concerning the current design, exist. However, under the current circumstances it is the most feasible method the assess data on the effectiveness of the intervention program.

## Competing interests

The authors declare that they have no competing interests.

## Authors' contributions

All authors critically reviewed the manuscript, read and approved the final manuscript. MG, MB, EvR, EC and JH are involved in the study design.

## Pre-publication history

The pre-publication history for this paper can be accessed here:

http://www.biomedcentral.com/1471-2318/10/11/prepub

## References

[B1] Hantikainen V Physical restraint: a descriptive study in Swiss nursing homes Nurs Ethics 1998 5 4 330 346 9782918 10.1177/096973309800500406

[B2] Hamers JP Huizing AR Why do we use physical restraints in the elderly? Z Gerontol Geriatr 2005 38 1 19 25 10.1007/s00391-005-0286-x 15756483

[B3] Meyer G Kopke S Haastert B Muhlhauser I Restraint use among nursing home residents: cross-sectional study and prospective cohort study J Clin Nurs 2009 18 7 981 990 10.1111/j.1365-2702.2008.02460.x 19284433

[B4] Halfens RJG Meijers JMM Neyens JCL Offermans MPW Rapportage resultaten Landelijke Prevalentiemeting Zorgproblemen 2007 2007 Maastricht: Universiteit Maastricht (Onderzoeksinstituut Caphri, Department of Healt Care and Nursing Sciences)

[B5] IGZ a Zorg voor vrijheid:terugdringen van vrijheidsbeperkende maatregelen kan en moet 2008 Den Haag: IGZ

[B6] Capezuti E Minimizing the use of restrictive devices in dementia patients at risk for falling Nurs Clin North Am 2004 39 3 625 647 10.1016/j.cnur.2004.02.015 15331306

[B7] Hamers JP Gulpers MJ Strik W Use of physical restraints with cognitively impaired nursing home residents J Adv Nurs 2004 45 3 246 251 10.1046/j.1365-2648.2003.02885.x 14720241

[B8] Werner P Perceptions regarding the use of physical restraints with elderly persons: comparison of Israeli health care nurses and social workers J Interprof Care 2002 16 1 59 68 10.1080/13561820220104177 11915718

[B9] Capezuti E Maislin G Strumpf N Evans LK Side rail use and bed-related fall outcomes among nursing home residents J Am Geriatr Soc 2002 50 1 90 96 10.1046/j.1532-5415.2002.50013.x 12028252

[B10] Capezuti E Strumpf NE Evans LK Grisso JA Maislin G The relationship between physical restraint removal and falls and injuries among nursing home residents J Gerontol A Biol Sci Med Sci 1998 53 1 M47 52 9467433 10.1093/gerona/53a.1.m47

[B11] Neufeld RR Libow LS Foley WJ Dunbar JM Cohen C Breuer B Restraint reduction reduces serious injuries among nursing home residents J Am Geriatr Soc 1999 47 10 1202 1207 10522953 10.1111/j.1532-5415.1999.tb05200.x

[B12] Evans D Wood J Lambert L Patient injury and physical restraint devices: a systematic review J Adv Nurs 2003 41 3 274 282 10.1046/j.1365-2648.2003.02501.x 12581115

[B13] Kron M Loy S Sturm E Nikolaus T Becker C Risk indicators for falls in institutionalized frail elderly Am J Epidemiol 2003 158 7 645 653 10.1093/aje/kwg203 14507600

[B14] Castle NG Mor V Physical restraints in nursing homes: a review of the literature since the Nursing Home Reform Act of 1987 Med Care Res Rev 1998 55 2 139 170 discussion 171-136 10.1177/107755879805500201 9615561

[B15] Miles SH Irvine P Deaths caused by physical restraints Gerontologist 1992 32 6 762 766 1478494 10.1093/geront/32.6.762

[B16] Gallinagh R Nevin R McAleese L Campbell L Perceptions of older people who have experienced physical restraint Br J Nurs 2001 10 13 852 859 11927885 10.12968/bjon.2001.10.13.852

[B17] Capezuti E Wagner LM Brush BL Boltz M Renz S Talerico KA Consequences of an intervention to reduce restrictive side rail use in nursing homes J Am Geriatr Soc 2007 55 3 334 341 10.1111/j.1532-5415.2007.01082.x 17341234

[B18] Evans LK Strumpf NE Allen-Taylor SL Capezuti E Maislin G Jacobsen B A clinical trial to reduce restraints in nursing homes J Am Geriatr Soc 1997 45 6 675 681 9180659 10.1111/j.1532-5415.1997.tb01469.x

[B19] Huizing AR Hamers JP Gulpers MJ Berger MP Short-term effects of an educational intervention on physical restraint use: a cluster randomized trial BMC Geriatr 2006 6 17 10.1186/1471-2318-6-17 17067376 PMC1635553

[B20] Koczy P Klie T Kron M Bredthauer D Rissmann U Branitzki S Guerra V Klein A Pfundstein T Nikolaus T [Effectiveness of a multifactorial intervention to reduce physical restraints in nursing home residents with dementia] Z Gerontol Geriatr 2005 38 1 33 39 10.1007/s00391-005-0289-7 15756485

[B21] Testad I Aasland AM Aarsland D The effect of staff training on the use of restraint in dementia: a single-blind randomised controlled trial Int J Geriatr Psychiatry 2005 20 6 587 590 10.1002/gps.1329 15920716

[B22] Wagner LM Capezuti E Brush B Boltz M Renz S Talerico KA Description of an advanced practice nursing consultative model to reduce restrictive siderail use in nursing homes Res Nurs Health 2007 30 2 131 140 10.1002/nur.20185 17380514

[B23] Huizing AR Hamers JP Gulpers MJ Berger MP Preventing the use of physical restraints on residents newly admitted to psycho-geriatric nursing home wards: A cluster-randomized trial Int J Nurs Stud 2009 46 4 459 69 10.1016/j.ijnurstu.2008.03.005 18486133

[B24] Huizing AR Hamers JP Gulpers MJ Berger MP A cluster-randomized trial of an educational intervention to reduce the use of physical restraints with psychogeriatric nursing home residents Journal of the American Geriatrics Society 2009 57 7 1139 1148 10.1111/j.1532-5415.2009.02309.x 19558484

[B25] Hamers JP Meyer G Kopke S Lindenmann R Groven R Huizing AR Attitudes of Dutch, German and Swiss nursing staff towards physical restraint use in nursing home residents, a cross-sectional study Int J Nurs Stud 2009 46 2 248 55 10.1016/j.ijnurstu.2008.06.007 18656876

[B26] Dunn KS The effect of physical restraints on fall rates in older adults who are institutionalized J Gerontol Nurs 2001 27 10 40 48 11820377 10.3928/0098-9134-20011001-10

[B27] Marek KD Rantz MJ Fagin CM Krejci JW OBRA '87: has it resulted in better quality of care? J Gerontol Nurs 1996 22 10 28 36 8954382 10.3928/0098-9134-19961001-13

[B28] Klink A Bussemaker J Fixatie en separatie 2008 Edited by Ministerie van Volksgezondheid WeS

[B29] Hamers JPH Gulpers MJM Reducing physical restraints in nursing homes: results of a pilot study J Nutr Health Aging 2009 Suppl 13 S17

[B30] Bartholomew LK Parcel GS Kok G Gottlieb NH Planning Health Promotion Programs: An Intervention Mapping Approach 2006 Hoboken: John Wiley & Sons

[B31] Janz NK Becker MH Stretcher VJ Glanz K, Rimer BK, Lewis FM The Heatlh Belief Model Health Behavior and Health Education: Theory, Research & Practice 2002 3 Hoboken: John Wiley & Sons

[B32] Arcares Registration of incidents and near accidents in nursing homes and old peoples' homes 2002 Utrecht: Arcares

[B33] Morris JN Fries BE Mehr DR Hawes C Phillips C Mor V Lipsitz LA MDS Cognitive Performance Scale J Gerontol 1994 49 4 M174 182 8014392 10.1093/geronj/49.4.m174

[B34] Morris JN Fries BE Morris SA Scaling ADLs within the MDS J Gerontol A Biol Sci Med Sci 1999 54 11 M546 553 10619316 10.1093/gerona/54.11.m546

[B35] Mahoney FI Barthel DW Functional Evaluation: the Barthel Index Md State Med J 1965 14 61 65 14258950

[B36] Cohen-Mansfield J Billig N Agitated behaviors in the elderly. I. A conceptual review J Am Geriatr Soc 1986 34 10 711 721 3531296 10.1111/j.1532-5415.1986.tb04302.x

[B37] de Jonghe JF Kat MG Factor structure and validity of the Dutch version of the Cohen-Mansfield Agitation Inventory (CMAI-D) J Am Geriatr Soc 1996 44 7 888 889 8675952 10.1111/j.1532-5415.1996.tb03762.x

[B38] Saunders RP Evans MH Joshi P Developing a process-evaluation plan for assessing health promotion program implementation: a how-to guide Health Promot Pract 2005 6 2 134 147 10.1177/1524839904273387 15855283

